# Recovery rate and associated factors of children age 6 to 59 months admitted with severe acute malnutrition at inpatient unit of Bahir Dar Felege Hiwot Referral hospital therapeutic feeding unite, northwest Ethiopia

**DOI:** 10.1371/journal.pone.0171020

**Published:** 2017-02-06

**Authors:** Hanna Demelash Desyibelew, Abel Fekadu, Haile Woldie

**Affiliations:** 1 Department Of Public Health, Faculty of Health Science, Woldia University, Woldia, Ethiopia; 2 Department of Epidemiology and Biostatistics, Institute of Public Health, College of Medicine and Health Sciences, University of Gondar, Gondar, Ethiopia; 3 Department of Human Nutrition, Institute of Public Health, College of Medicine and Health Sciences, University of Gondar, Gondar, Ethiopia; Institut de recherche pour le developpement, FRANCE

## Abstract

**Background:**

Despite numerous advances made in improving child health and the clinical management protocols for treating severe acute malnutrition at treatment centers, evidences concerning the treatment outcomes are scarce. Therefore, this study was conducted to assess the recovery rate and associated factors of severely acute malnourished children of age 6 to 59 months admitted to inpatient therapeutic feeding unit at Felege Hiwot Referral Hospital.

**Methods:**

We conducted a hospital-based cross-sectional study including 401 severely malnourished children who were admitted from September 2012 to January 2016. Bivariable and a Multivariable logistic regression model were fitted to identify factors associated with recovery rate. Adjusted Odds ratio with its 95% CI was reported and P-value less than 0.05 was considered as significant.

**Results:**

Fifty eight percent (58.4%) (95%CI: 53.1–64.1) of admitted children were recovered with a mean recovery time of 18 (±6.3) days. Being female, children who were fully and partially vaccinated, who had better MUAC measurement, who stayed longer in the hospital, and children who took routine vitamin-A supplementation had better recovery rate. However, children who had co-morbidity at admission, had human immune virus (HIV) and Tuberculosis (TB) infection, and who had edema were less likely to recover.

**Interpretation:**

Recovery rate was low as compared to international SPHERE cutoff points (> 75% recovery rate). Interventions that could address the outlined factors would be helpful to improve treatment recovery rate of admitted children.

## Background

Severe acute malnutrition (SAM) is defined by a very low weight for height, by visible severe wasting or by the presence of nutritional edema. In children aged 6–59 months, mid upper arm circumference(MUAC) less than 110 mm or 11cm is also indicative of severe acute malnutrition [1,2].

Despite numerous advances made in improving child health, severe acute malnutrition remains the major killer of children under five years of age. These high burdens of child mortality due to severe acute malnutrition remain a problem for the last couple of years. However, few countries in high prevalence areas have specific national policies aimed at addressing severe acute malnutrition comprehensively [1,3].

Globally, around 1 to 2 million children die every year due to severe acute malnutrition and 20 million children live with severe acute malnutrition [1,4]. In developing countries around 2% of children have SAM; from this, South Asia and Sub-Saharan African countries shoulder more [4]. SAM is the commonest cause of hospital admission in the pediatric ward and it is a reason for 25 to 30% death in many poor countries [1,4,5].

Ethiopia is one of the countries in the sub-Saharan Africa with the highest rates of severe acute malnutrition. Over the past fifteen years, the trend of malnutrition revealed that there is a reduction in stunting by 31% and underweight by 39%. However, there was only a small decline in the prevalence of wasting over the last 15 years (from 12% to 9%). In Ethiopia, 3% of under-five children have SAM and 2.7% are found in Amhara region [6]. Locally, in Bahir Dar town, among malnourished under five years children, 24.8% were due to SAM [7].

Similarly, SAM is the primary diagnosis in 20% of pediatric hospital admissions in Ethiopia [8]. The problem of SAM is not only medical disorder rather it is also social disorder. Therefore, successful management of severely malnourished patients requires both medical and social efforts. Child under-nutrition has long-term negative effects on child’s lives and this will affects the human capital of a country on which the economy relies [2,6,8]. In Ethiopia, the annual costs associated with child under-nutrition are estimated to be US$ 4.7 billion, which is equivalent to 16.5% of GDP [8].

Few studies on recovery rate in the hospitals treating SAM of under five years children in Ethiopia remained below the acceptable cutoff point[4,7,9,10]. The limited evidences on the area so far made the researchers to investigate the recovery rate of SAM based on the sphere standard recovery rate [11]. This would help to improve management of SAM thereby reducing the associated burden of the disease.

## Methods

### Study setting, design, and participant

Bahir Dar is found at a distance of 563 kilometers away from Addis Ababa and located on the Southern shore of Lake Tana, the source of the Blue Nile. In Bahir Dar city administration, for the total of 31,800 populations, there are 2 hospitals, 10 health centers, 10 health posts, and 134 private health institutions.

This study was conducted at Feleg Hiwot Referral hospital, which is the referral hospital for the region, located in the capital city of the region. The Pediatric department is among the five inpatient departments in the hospital. The outpatient treatment center was established and has been giving the service since 2002.

Ethiopia adopted a set of guidelines from WHO SAM guideline protocol for inpatient management of severe malnutrition in children under 5 years of age. The guideline divides the management in three phases; the first phase (phase I), transition phase, and phase II. In phase I, admitted SAM children resuscitated, treated for infections, restored electrolyte balance, and prevented hypoglycemia and hypothermia on indication. F75 (130ml = 100kcal) milk therapeutic food was used during phase I treatment. Malnourished cases who responded on treatment by return of appetite, beginning of loss of edema, and no intravenous line or nasogastric tubes were transferred to transition phase to receive F100 (diluted F100, 130ml = 130kcal).

A sudden change to large amounts of diet (from F75 to F100) before physiological function restored can be dangerous and lead to electrolyte disequilibrium, so, to prevent this, F100 diluted is given at transition phase. The quantity of F100 diluted given is equal to the quantity of F75 given in Phase I but less than the quantity given at phase II. It is made of one large package of F100 diluted into 2 liters of water or one small package diluted into 500 ml of water.

Afterwards, cases were transferred to phase II after they gained good appetite and clear edema. In phase II treatment, F100 was used as much as the children could take and additional diet is recommended until they achieved weight for height > 85% and no edema for 10 consecutive days. In addition, Routine medicines of vitamin-A, folic acid, iron, antibiotics, anti-malaria, measles, and deworming was used in the treatment of SAM children[2].

### Study design and period

A hospital based cross-sectional study using a retrospective record review was conducted from February 25 to March 15, 2016. All 6 to 59 months of severely acute malnourished children who were admitted and treated at an inpatient therapeutic feeding units from September 2012 to January 2016 were included in the study.

### Sample size and sampling procedure

We determined the minimum sample size using a single population proportion formula [n = [(Z_a/2_)^2^*P (1-P)]/d^2^] by assuming 95% confidence level (Z a/2 = 1.96), margin of error 5%, recovery rate of 85%, and a 10% addition for missing/incomplete data. The final sample size was 215 for the first objective. We used two population proportion formulas to determine a sample size for the second objective that is risk factor identification. Accordingly, a sample size of 374 was calculated, but, when the four years multi-chart was reviewed, 401 records were there and all were considered for analysis.

### Data collection instruments and procedure

Pretested data abstraction sheet was adopted from Federal Ministry of Health SAM management guideline to address the study variables. We provided a two days training for six data collectors and two supervisors for two days. Investigators coordinated the overall activities of data collection. Collected data were sorted and checked for errors and completeness before analysis.

### Variable definition

Recovery was considered when the admitted children with weight less than 70% achieved the target weight ≥85% of weight for height or weight for length at discharge. On top of that, edema for consecutive 10days should not be observed[2]. This target weight is calculated by using the NCHS/WHO normalized chart.

Recovery rate is calculated as a number of SAM children discharged after recovery divided by the total number of SAM children admitted at TFU[2].

Kwashiorkor is severe under nutrition or malnutrition in children resulting from a diet excessively high in carbohydrates and low proteins.

Marasmus is an extreme under nutrition or malnutrition and emaciated that from inadequate intake of carbohydrate food or from mal-absorption or metabolic disorders.

Marasmic-kwashiorkor is the mixture of both kwashiorkor and marasmus. So, it is due to both carbohydrate and protein containing food deficiency [2].

### Data processing and analysis

Data were checked, coded and entered into Epi-Data version 3.1 and was exported to SPSS version 16 for analysis. The data were checked for missingness and fulfillment of assumptions was checked by using frequencies and cross tabulations.

Bivariable analyses were done and all covariate variables which had an association with the outcome variables at p-value of 0.25 were entered in to multivariable model. A two-sided P value ≤0.05 was considered to indicate statistical significance. The strength of association was expressed in odds ratio (OR). The assumption of the goodness of the model was checked by using Hosmer—Lemeshow goodness of fit test and backward logistic regression method was used to select significant variables.

## Result

### Socio-demographic characteristics

A total of 401 children with severe acute malnutrition were admitted to therapeutic feeding units from September 2012 to January 2016. Out of these reviewed records, 223 (55.6%) were males and 178 (44.4%) were females. Similarly, among the recovered 234 SAM children, 118(50.4%) were males and 116(49.6%) were females. The mean age of these children was 23.9 (±14.8) months. (Table 1)

**Table 1:** Distribution of socio-demographics characteristics among SAM children Northwest, Ethiopia, 2016 (n = 401).

Variables	Recovered Frequency (%)	Not recovered Frequency (%)	Total (n = 401) Frequency (%)
**Sex**			
Male	118 (50.4)	105 (62.9)	223 (55.6)
Female	116 (49.6)	62 (37.1)	178 (44.4)
**Age**			
6–24	149 (63.7)	106 (63.5)	255 (63.6)
25–59	85 (36.3)	61 (36.5)	146 (36.4)
**Residence**			
Urban	70 (29.9)	55 (32.9)	125 (31.2)
Rural	164 (70.1)	112 (67.1)	276 (68.8)
**Breast feeding**			
Yes	226 (96.6)	153 (91.6)	379 (94.5)
No	8 (3.4)	14 (8.4)	22 (5.5)
**Vaccination for age**			
Unvaccinated	10 (4.3)	32 (19.2)	42 (10.5)
Partial vaccinated	19 (8.1)	13 (7.8)	32 (8)
Full vaccinated	205 (87.6)	112 (73.1)	327 (81.5)

### Anthropometry and type of malnutrition

The descriptive baseline results showed that a recovered child had a mean weight of 8.4kg (±6.2Kg) and a mean MUAC of 11.3cm (±1.2cm) at discharge. Recovered edematous children had the longest mean length of stay which was 19days (±6.5 days) and the highest mean weight of 9.9kg (±2.5Kg). Mean length of stay for severely wasted children was 17days (±6days) and mean weight for severely wasted children was 7.6kg (±2.3Kg).

More than half (63.1%) of the children enrolled into the study had severe wasting and 36.9% had edema (kwashiorkor or marasmic kwashiorkor).

### Recovery rate from SAM

Of 401 admitted children whose records was reviewed, 234 (58.4%) were recovered (95%CI: 53.1–64.1). From the total of 401 children admitted to therapeutic feeding units, 308 (78.4%) were recovered from phase I, whereas 285 (89.3%), and 233 (81.5%) were recovered at transition phase and phase II, respectively. (Table 2)

**Table 2:** Program performance indicators of children with SAM admitted to TFU, Northwest Ethiopian (N = 401).

Variables	Phase I Number (%)	Transition Number (%)	Phase II Number (%)	Total Number (%)
Recovered	308 (78.4)	285 (89.3)	233 (81.5)	234 (58.4)
Defaulter	52 (13.2)	44 (12.9)	15 (5.2)	87 (21.7)
Death	31 (7.7)	5 (1.5)	0	34 (8.5)
Transfer outs	1 (0.3)	3 (0.9)	31 (10.8)	35 (8.7)
Non-responder	1 (0.3)	4 (1.3)	7 (2.4)	11 (2.7)

The mean (±SD) recovery time of SAM children was 18 days (±6.3), whereas for wasted and edematous children, it was 17 days (±6) and 19 days (±6.5), respectively.

### Co-morbidity

Ninety percent (90.5%) of under-five years SAM admitted children at the therapeutic feeding units had at least one form of co-morbidities. Likewise, the majority of wasted (92.9%) and edematous (86.5%) SAM children had co-morbidities at admission. The rest 7.1% of wasted and 13.5% of edematous children were admitted with only the diagnosis of severe malnutrition. Diarrhea (36.2%), pneumonia (39.2%), anemia (29.7%s), and gastrointestinal tract infections (29.4%) were the prevalent comorbidities. (Table 3)

**Table 3:** Co-morbidity patterns among children With SAM admitted to TFUs, Northwest Ethiopia 2016.

Variables	Category	Recovered N (%)	Not recovered N (%)	Total number (n = 401) N (%)
Diarrhea	Yes	88 (37.6)	57 (34.1)	145 (36.2)
	No	146 (62.4)	110 (65.9)	256 (63.8)
TB	Yes	10 (4.3)	27 (16.2)	37 (9.2)
	No	224 (95.7)	140 (83.8)	364 (90.8)
HIV	Yes	5 (2.1)	75 (44.9)	26 (6.5)
	No	229 (97.9)	92 (55.1)	375 (93.5)
Pneumonia	Yes	82 (35.0)	75 (44.9)	157 (39.2)
	No	152 (65.0)	92 (55.1)	244 (60.8)
URTI	Yes	16 (6.8)	18 (10.8)	34 (8.5)
	No	218 (93.2)	149 (89.2)	367 (91.5)
Anemia	Yes	70 (29.9)	49 (29.3)	119 (29.7)
	No	164 (70.1)	118 (70.7)	283 (70.3)
GTI	Yes	73 (31.2)	45 (26.9)	118 (29.4)
	No	161(68.8)	122 (73.1)	283 (70.6)
Malaria	Yes	5 (2.1)	8 (4.8)	13 (3.2)
	No	229 (97.9)	157 (95.2)	388 (96.8)
CHF	Yes	7 (3.0)	22 (13.2)	29 (7.2)
	No	227 (97.0)	145 (86.8)	372 (92.8)
Rickets	Yes	19 (8.1)	23 (13.8)	42 (10.5)
	No	215 (91.9)	144 (86.2)	359 (89.5)
Sepsis	Yes	8 (3.4)	12 (7.2)	20 (5.0)
	No	226 (96.6)	155 (92.8)	381 (95.0)
Developmental delay	Yes	15 (6.4)	10 (6)	25 (6.2)
	No	219 (93.6)	157 (94)	376 (93.8)
Others*		5 (2.1)	23 (13.8)	28 (6.9)

*others otitis media, crop, refeeding diarrhea, Kwashdermatitis

### Management protocols

Management of admitted cases with severe acute malnutrition in the therapeutic feeding unit (TFU) was done in accordance with the national guidelines for treatment of SAM. This study result revealed that 90.5% and 47.6% of SAM admitted children were taken routine and special medications, respectively during their therapeutic feeding unit stay.

According to the Ethiopian SAM inpatient management protocol, 363 (90.5%) children did receive routine medications partially. However, 38 (9.5%) eligible children did not receive any type of routine medications. The most commonly administered routine medications were ampicillin and gentamycin (75.6%), vitamin-A (71%), and folic acid (95%). Children who presented with diarrhea were also received Zinc (28.8%) in addition to routine medications.

On the other side, the commonly used therapeutic foods were F75 (98%), F100 dilute (78.6%), and F100 (68%). Almost all SAM admitted children had a similar frequency (6 times per day) of therapeutic food at each phase. (Table 4)

**Table 4:** Distribution of treatments and feedings given to 6 to 59months of children With SAM admitted to TFU, Northwest Ethiopia, 2016 (n = 401).

Variables	Recovered N (%)	Not recovered N (%)	Total (n = 401) N (%)
**Routine medication**			
Yes	229 (97.9)	158 (94.6)	387 (96.5)
No	5 (2.1)	9 (5.4)	14 (3.5)
**Vitamin—A**			
Yes	188 (80.3)	97 (58.1)	285 (71.1)
No	46 (19.7)	70 (41.9)	116 (28.9)
**Folic acid**			
Yes	225 (96.2)	156 (93.4)	381 (95)
No	9 (3.8)	11 (6.6)	20 (5)
**Amoxicillin**			
Yes	63 (26.9)	34 (20.4)	97 (24.2)
No	171 (73.1)	133 (79.6)	304 (75.8)
**Ampicillin and gentamycin**			
Yes	176 (75.2)	127 (76)	303 (75.6)
No	58 (24.8)	40 (24)	98 (24.4)
**Measles**			
Yes	20 (8.5)	10 (6)	30 (7.5)
No	214 (91.5)	157 (94)	371 (92.5)
**Iron**			
Yes	48 (20.5)	25 (15)	73 (18.2)
No	186 (79.5)	142 (85)	328 (81.8)
**Deworming**			
Yes	57 (24.4)	26 (15.6)	83 (20.7)
No	177 (75.6)	141 (84.4)	318 (79.3)
**Special medication**			
Yes	91 (38.9)	100 (59.9)	191 (47.6)
No	143 (61.1)	67 (40.1)	210 (52.4)
**Special medication type**			
Antibiotics	29 (31.9)	36 (36)	65 (16.2)
Zink	38 (16.2)	17 (17)	55 (13.7)
Vitamin-D	6 (6.6)	4 (4)	10 (2.5)
Others*	18 (19.8)		61 (15.2)
**F75**			
Yes	229 (97.9)	164 (98.2)	393 (98)
No	5 (2.1)	3 (1.8)	8 (2)
**F100**			
Yes	222 (94.9)	51 (30.5)	273 (68.1)
No	12 (5.1)	116 (69.5)	128 (31.9)
**Plumpy’nut**			
Yes	7 (3)	2 (1.2)	9 (2.2)
No	227 (97)	165 (98.8)	392 (97.7)

*Lasix’s, minerals and vitamins other than vitamin-D and zinc

### Factors associated with recovery rate

After adjusting for socio-demographic, economic, type of medication, and other co-morbidities; sex, vaccination for age, routine medication (vit-A), co-morbidities at admission, TB, HIV, edema, MUAC, and length of stay had significant association with nutritional recovery rate of SAM children.

Accordingly, those children who were fully (AOR: 4.12; 95%CI: 1.64–10.35) and partially (AOR: 7.16; 95%CI: 1.97–25.25) vaccinated for age had better recovery rate than those children who hadn’t been vaccinated. Compared to male, females were 86% times more likely to recover (AOR: 1.86; 95%CI: 1.11–3.11).

Edematous children were less likely to be recovered than wasted children (AOR: 0.46; 95%CI: 0.22–0.95). Recovery rate was increased by 44% for every one-centimeter increase in MUAC (AOR: 1.44; 95%CI: 1.09–1.90). The overall length of stay for the entire cohorts of children with SAM was significantly associated with recovery rate; for a one day increase in stay in the therapeutic feeding units, the recovery rate would increase by 9% (AOR: 1.09;95%CI:1.05–1.12).

At admission, children who were presented with co-morbidity were 84% times less likely to be recovered than children without co-morbidities at admission (AOR: 0.16; 95%CI: 0.05–0.51). Severely malnourished children co-morbid with HIV/AIDS (AOR: 0.12; 95%CI: 0.03–0.41) and tuberculosis (AOR = 0.13; 95%CI: 0.04–0.35) were less likely to be recovered. Lastly, children who did take vitamin-A as routine medication were 2.8 times more likely to be recovered as compared to those who did not take vitamin-A (AOR = 2.84;95%CI:1.41–5.72). (Table 5)

**Table 5:** The bivariable and multivariable logistic regression of factors associated with nutritional recovery rate from SAM at Felege hiwot TFU Northwest, Ethiopia 2016 (n = 401).

Variables	Recovered Frequency (%)	Not recovered Frequency (%)	COR (95% CI)	AOR (95% CI)
**Sex**				
Male	118 (50.4)	105 (62.9)	1	1
Female	116 (49.6)	62 (37.1)	1.66 (1.11–2.49)	1.86 (1.11–3.11)*
**Vaccination for age**				
Unvaccinated	10 (4.3)	32 (19.2)	1	1
Partial vaccinated	19 (8.1)	13 (7.8)	4.68 (1.72–12.72)	7.06 (1.97–25.25)*
Fully vaccinated	205 (87.6)	112 (73.1)	5.38 (2.55–11.32)	4.12 (1.64–10.35)*
**Breastfeeding**				
Yes	226 (96.6)	153 (91.6)	2.58 (1.06–6.31)	
No	8 (3.4)	14 (8.4)	1	
**Edema**				
Yes	78 (33.3)	70 (41.9)	0.69 (0.46–1.04)	0.46 (0.22–0.95)*
No	156 (66.7)	97 (58.1)	1	1
**Admission status**				
New	212 (90.6)	146 (87.4)	1.00 (0.42–2.41)	
Readmission	9 (3.8)	12 (7.2)	0.52 (0.15–1.74)	
After default	13 (5.6)	9 (5.4)	1	
MUAC	234 (58.4)	167 (41.6)	1.45 (1.24–1.73)*	1.44 (1.09–1.90)*
Length of stay in TFU	234 (58.4)	167 (41.6)	1.08 (1.05–1.11)*	1.09 (1.05–1.12)*
**Co-morbidity**				
Yes	201 (85.9)	162 (97)	0.19 (0.07–0.49)*	0.16 (0.05–0.51)*
No	33 (14.1)	5 (3)	1	1
**Pneumonia**				
Yes	82 (35)	75 (44.9)	0.66 (0.44–0.99)*	
No	152 (65)	92 (55.1)	1	
**Tuberculosis**				
Yes	10 (4.3)	27 (16.2)	0.23 (0.11–0.49)*	0.13 (0.04–0.35)*
No	224 (95.7)	140 (83.8)	1	1
**HIV/AIDS**				
Yes	5 (2.1)	21 (12.6)	0.15 (0.056–0.41)*	0.12 (0.03–0.41)*
No	229 (97.9)	146 (87.4)	1	1
**Vitamin-A**				
Yes	188 (80.3)	97 (58.1)	2.95 (1.89–4.60)	2.84 (1.41–5.72)*
No	46 (19.7)	70 (41.9)	1	1
**Folic acid**				
Yes	225 (96.2)	156 (93.4)	1.76 (0.71–4.35)	
No	9 (3.8)	11 (6.6)	1	
**Deworming**				
Yes	57 (24.4)	26 (15.6)	1.74 (1.04–2.92)	
No	177 (75.6)	141 (84.4)	1	
**Amoxicillin**				
Yes	63 (26.9)	34 (20.4)	1.44 (0.89–2.32)	
No	171 (73.1)	133 (79.6)	1	
**Special medication**				
Yes	91 (38.9)	100 (59.9)	0.42 (0.28–0.64)	
No	143 (61.1)		1	

*significant association at p-value <0.05

## Discussion

Though, numerous advances were made to improve child survival, severe acute malnutrition is a global problem especially in developing countries like Ethiopia [1,3]. The present study assessed a record of 401 in patients of age 6 to 59 months children who were managed according to the severe acute malnutrition national treatment guideline [2].

This study revealed that the recovery rate was 58.4%, which was lower than the international standard in which the minimum recovery rate is greater than 75% [11]. This finding is also lower than the study conducted in Woldia hospital, north Ethiopia which showed 85% recovery rate [12]; Southern Ethiopian which showed 82.4% recovery rate [13]; and Jimma University Specialized hospital (JUSH), which showed 77.8% recovery rate [9]. However, this study is greater than a studies from south Asian countries; a 51.7% recovery rate in India [14]; and a 50% recovery rate in Pakistan[15]. This indicates that a recovery rate from severe acute malnutrition in these children is low, which could be considered as a major challenge for the program in the future.

The possible reasons for low recovery rate in this study as compared to other studies could be attributed to unacceptable higher defaulter rate in the current study compared to the previous studies. On top of that, fail to comply with the national SAM management standard protocol, like most routine medications administration, might be other possible reason. Lastly, since the study setup was at the referral hospital, patient overload and high burden of comorbidities like chronic diseases (CHF, TB) might also account for the low recovery rate in the current study.

Similar to a retrospective cohort study reported from southern Ethiopia [13], socio-demographic characteristics like age, residence, and breastfeeding were not statistically associated with recovery rate. However, child vaccination status and female sex have showed statistically significant association with recovery rate. In this study finding, being female had 1.8 times higher probability of getting recovered as compared to being male which is inconsistent with the finding published in Bangladesh[16]. The possible reason for the inconsistency might be due to the difference in inpatient management guideline at entry or discharge that has been used in the two countries.

The probability of recovery was reduced by 84% in children who had co-morbidities at admission. In agreement with this finding, a study from Jimma university specialized hospital reported less recovery and a higher probability of death in children with co-morbidity[9]. The possible reason might be a child with co-morbidities needs a longer hospital stay, increased nutrient loss, and more nutrient requirement with decreased nutrient absorption and utilization as compared to malnourished children in the absence of co-morbidity.

In agreement with this finding, Collins et al. reported that SAM children presented with HIV/AIDS and TB at admission were less likely to be recovered as compared to SAM children without HIV/AIDS and TB [17]. Similarly, children with HIV/AIDS in this study were less likely to recover than those who were not, in contrast to a study conducted in Woldia hospital, North Ethiopia[12] and Zambia [18,19].

According to the finding of this study, SAM children who had edema were less likely to be recovered as compared to severely wasted children without edema, which is in line with the finding from India[14] and northern Ethiopia[12]. However, it is not consistent with studies reported from Southern Ethiopia [13], Bangladesh [16], and Zambia [19] that reported edematous children had better recovery rate as compared to severely wasted children. The possible reason might be the fact that edematous children were prone to develop a metabolic complication and fluid overload with chronic diseases like chronic heart failure. The other possible reason might be, children with edema are more ill; therefore their condition is more severe and would take longer time to recover.

Likewise, the overall mean length of stay in the TFU that was 15.4 days was consistent with the minimum international standard set for management of SAM (less than 28 days) [11] and with other studies [16,20]. However, the overall length of stay in the current TFU was lower than the other studies mean length of stay [9,12,13,18,19]. Higher recovery rate and lower defaulter rate achieved in these studies might be attributed to their longer stay in hospital.

The current study finding revealed that the nutritional recovery rate of children with SAM was significantly increased as the length of stay or MUAC of the children increased. For every additional one day stay in the therapeutic feeding units, the nutritional recovery rate was increased by 9% which is in line with studies from Southern Ethiopia [13]. The possible reason might be the more the child stay at therapeutic feeding units the less to default and have more chance to complete nutritional therapy and more likely to have a better outcome.

Similarly, for every one centimeter increase in MUAC of the children, the nutritional recovery rate was increased by 44%. This study finding is consistent with other similar studies [13,21,22]. This might be due to the fact that early detection and admission with less severe wasting would increase the probability of better treatment outcome. Similarly, severely malnourished children who have received routine medication like vitamin-A at admission or discharge or both at admission and discharge were 2.8 times more likely to recover as compared to children who did not receive vitamin-A. As a limitation, since the study fully relied on children’s secondary data, it is impossible to include essential covariates that could more explain the association.

## Conclusion and recommendation

This retrospective multi-chart record review revealed that recovery rate is remaining low as compared to a standard sets in humanitarian and disaster prevention (or the SPHERE standards) and most studies that were done in Ethiopia.

According to this study, being female, vaccinated for age, having better MUAC measurement, longer length of stay, and taking vitamin-A routine supplement had increased the odds of recovery. However, having edema and co-morbidities at admission, TB, and HIV infection decreased the odds of recovery of SAM children admitted at inpatient therapeutic feeding units. Intervention modalities that would address the identified factor are highly recommended for better recovery rate achievements.

## Declarations

### Ethics approval and consent to participate

Ethical clearance was obtained from the ethical review committee of University of Gondar, College of medicine and health science. Additionally, permission letter was also secured from Bahir Dar Felegehiwot Referral Hospital. Since the study was based on secondary data there was no direct contact with patients. So anonymity was maintained by using identity numbers instead of patient names. Besides, all the data abstracted was kept confidential and not used for any other purposes than the stated research objective.

### Consent for publication

Consent for publication is secured from study participants.

## Abbreviations

95% CI: 95% Confidence Interval
AOR: Adjusted odds ratio
CHF: Chronic Heart Failure
COR: Both Crude Odds Ratio
HIV/AIDS: Human Immune Virus/ Acquired Immune Deficiency Syndrome
JUSH: Jimma University Specialized Hospital
MUAC: Mid-Upper Arm Circumference
SAM: Severe Acute Malnutrition
SPSS: Statistical package for social sciences
TB: Tuberculosis
TFU: Therapeutic Feeding Units
Vit-A: Vitamin-A
WHO: World Health Organization
